# The effect of diabetes complications on health-related quality of life: estimating the bias due to unobserved heterogeneity using the UKPDS

**DOI:** 10.1186/1745-6215-12-S1-A64

**Published:** 2011-12-13

**Authors:** Maria L Alva, Alastair Gray, Borislava Mihaylova, Philip Clarke

**Affiliations:** 1 HERC, Department of Public Health, University of Oxford, Oxford, UK; 2 Sydney School of Public Heath, University of Sydney, Australia

## 

The impact of six diabetes related complications: myocardial infarction, ischemic heart disease, stroke, heart failure and amputation on quality of life is studied based on EQ-5D data on 3,380 patients collected in the UKPDS Post Trial Monitoring Study over the period 1997-2007. Analysts tend to resort to cross-section data to estimate the determinants of quality of life, as these are easier and less expensive to collect. The use of cross-section data may come at a cost in terms of biased estimators due to unobserved heterogeneity that can be addresses by the use of panel data.

The importance of non response (e.g. sample selection) is tested, and cross-sectional and longitudinal approaches to study effects on QoL are compared. The variation in utility across patients in our data is more than twice that observed within a patient over-time and, this heterogeneity is often correlated with the likelihood of having complications, making the results of pooled estimations biased. Cross-sectional analysis in our sample over-estimates the effects of complications on utility. We address this problem by using fixed effects (FE). The bulk of the variation in the unexplained utility appears to be largely determined by time-invariant differences across patients’ characteristics: gender, race, socio economic status and habits. We therefore link panel data methods with cross sectional analysis in a two-stage analysis, using the fixed effects as the dependent variable in the second stage regression to examine the extent to which certain time-invariant patient characteristics and variables recorded only at baseline in the trial, are related to patients’ utility score. The results highlight the importance of studying quality of life changes over time to distinguish between time invariant correlates of wellbeing and the effect of diabetes complications.

